# The effects of pricing policy on the prices and supply of low-cost medicines in Shandong, China: evidence from an interrupted time series analysis

**DOI:** 10.1186/s12889-020-08746-x

**Published:** 2020-04-29

**Authors:** Xuejing Rong, Jia Yin, Shuoyun Duan, Qiang Sun, Zaheer-Ud-Din Babar

**Affiliations:** 1 School of Health Care Management, Shandong University, Jinan, 250012 Shandong China; 2 NHC Key Laboratory of Health Economics and Policy Research (Shandong University), Jinan, 250012 Shandong China; 3 Centre for Pharmaceutical Policy and Practice Research, Department of Pharmacy, University of Huddersfield, Queensgate, Huddersfield, HD1 3DH UK

**Keywords:** Essential medicine, Supply, Medicine shortages, Pricing policy, Interrupted time series, Centralized procurement, Delivery time

## Abstract

**Background:**

In China, some medicines had a supply problem. In 2015, to address this problem, the Chinese government issued a policy to raise the price cap for some shorted low-cost medicines (LCMs). The objective was to assess the effects on medicine prices and supply of medicines from a medicine pricing policy reform point of view.

**Methods:**

This study was conducted in Shandong, an eastern province of China with a population of 99.4 million. We collected procurement data of all (*n* = 1494) LCM medicines available between April 2014 and February 2017 from the web-based Provincial Drug Centralized Bidding Procurement System. This study used the Drug Price index and the average price to reveal the price change of LCMs and used the interrupted time series to evaluate the effects of LCM policy on medicine supply by measuring the change of monthly procurement volume, the number of products, and the average delivery time of LCMs.

**Results:**

After the policy implementation in October 2015, the quarterly average price of all LCM products, especially traditional Chinese medicines, showed a sudden growth trend. Then after two-quarter implementation of policy, the price recovered to the same trend before policy intervention, which is consistent with the trend of the Drug price index. There were 466 of LCM products available in October 2015. After the policy intervention, the number of products available increased by 109.87% (*n* = 978) in February 2017, at a growth rate of 6.44% per month (Value = 30.02, *P* < 0.001). Besides after the intervention in October 2015, the monthly procurement volumes of LCMs increased rapidly, on average, at a rate of 28.93% per month (Value = 474,000, *P* < 0.001) for all LCMs. The average delivery time of LCMs kept on decreasing from 33.37 days to 10.69 days at a reduced rate of 3.63% (Value = − 1.21, *P* < 0.001) per month before the policy, while no significant changes were noted. Also, average monthly delivery time was stable at 9 days after the intervention.

**Conclusions:**

The policy promoted the supply of low-cost medicines, which is beneficial for the Universal Health Coverage. However, future policies should focus on monitoring price change and reducing the delivery time of generic medicines.

## Background

Globally, medicine shortage is a big challenge for achieving Universal Health Coverage (UHC) [1–4]. China is also facing the problem of medicines shortage. A study conducted in Shandong province found that only 69% of the essential medicines were available in hospital pharmacies in 2006 [5]. A survey in Shaanxi Province illustrated that mean availability of surveyed medicines was low in both the public and private sectors; availability of lowest-priced generics declined from 27·4% to 22·3% during 2010–2012, particularly in primary hospitals [6]. A qualitative study in China reported that 95 medicines (of which, 51 were essential medicines) were out of supply in 2015, owing to the problems of manufacturing, distribution and supply. All these medicines had quite low unit prices [7]. This problem is typical for generic medicines [8, 9] because long-term unchanged maximum retail price caps might make some generic medicines’ prices set at an unprofitable level and pharmaceutical companies earned no benefit to producing them in China before 2015 [7, 10–15].

In response to generic medicine shortage problem, China’s National Development and Reform Commission (NDRC) and National Health Commission (NHC) issued a new pricing policy to raise the price cap of some low-cost medicines (LCMs) in 2015 [16]. The NDRC selected 533 medicine by chemical names of medicines and stipulated that the prices of those medicines cannot be higher than the maximum daily cost (RMB 3 for chemical drugs and RMB 5 for Chinese traditional medicines) according to the average daily dosage on drug labels national wide [17]. In the Low-cost medicines list, about one-third of those medicines are essential medicines. Meanwhile, each province would add medicines to the LCMs list according to their demands. For example, Shandong province’s supplementary low-cost medicines list has 210 medicines by chemical names and there are 743 LCMs by chemical names in Shandong [18]. Therefore, the final LCMs lists for each province varies in China. The policy [16] aim was to promote the supply of LCMs and to satisfy the demand for generic medicines.

The transformation of the price cap of LCMs has been implemented for some time in China and studies about this policy are mainly focused on price analysis. Zhang (2016) [19] Song (2018) [20] and Guan (2018) [21] all found that the price of LCMs increased after the policy implementation. Furthermore, from the patients’ perspective, Wang (2017) [22] found that the patients’ awareness of LCMs is quite low and patients’ satisfaction with the policy needs to be further improved. Additionally, Duan (2019) [23] evaluated the effects of LCM policy on purchasing of chemical medicines only and found that the policy increased the purchasing volume. However, little empirical evidence was available to provide a rounded and comprehensive analysis of the effects of the ceiling prices changes on the price as well as the supply of all LCMs. This study aims to fill this gap. The study objective was to analyze the effects of LCM policy on medicine prices, availability and supply in Shandong province in China.

## Methods

### Study setting and design

This study was conducted in Shandong, an eastern province of China. All secondary and tertiary public hospitals (not include primary health institutes) in Shandong started implementing LCM policy in October 2015. We choose this province because we have all the LCMs selling records of Shandong public hospitals. In 2017, Shandong Province had a population of 99.4 million, ranking second thickly populated among 31 provinces in China. In 2017, the GDP was 72,63.42 billion RMB in Shandong which ranked third among all 31 provinces (including four independent municipalities) in China and the average GDP per capita is RMB 72,580.60, ranked 8th among all provinces in China. (National Bureau of Statistics of China, 2017).

This is a retrospective study. The first part of this study was to analyze the price change as an impact of LCM policy. We set our study period started from 2nd quarter 2014 to 1st quarter 2017 and used the quarterly average price to show the trend of all LCMs. Besides, we selected 334 LCM products that have demand from hospitals every quarter to calculate Drug price index and to display the trend of LCMs prices. By comparing those two indicators, we analyzed how prices of LCMs changed with the passage of time (before and after the policy).

The second part is to analyze the policy effects on medicines supply. We used the single interrupted time series (ITS) analysis to evaluate the longitudinal effectiveness of LCM policy [24, 25]. Interrupted time series analysis could validate whether the implementation of the policy has an effect significantly greater than the underlying trend by collecting data at multiple instances overtime before and after the policy intervention [26, 27]. The intervention of our study is the LCM policy implemented in October 2015. Our interrupted time series study period was from April 2014 to February 2017. There were 19 months before the intervention and 16 months after the intervention. The outcome measures were monthly procurement volumes, the number of products, and the average delivery time of LCMs (explained below).

### Outcome measures in ITS

#### Monthly number of products

The monthly number of products means the number of LCM products with different chemical names, dosage forms and specifications (not consider brands) available each month. If the monthly number of products increases, it means there are more LCMs available from pharmaceutical companies than before. The change of this indicator could reflect the supply of LCMs. This indicator is from the supply perspective.

#### Monthly procurement volumes

The monthly procurement volumes mean the volumes of LCMs purchased by hospitals every month and this could reflect the demand for hospitals. However, the sales volumes of total LCMs procured could be influenced by the sales volumes of newly supplied LCMs as well as the sales volumes of the existing LCMs. We selected 154 LCMs among 743 LCMs by chemical name that were purchased every month during our study period and this indicator could reflect the policy effect on LCMs procurement without the biases from medicine types. If this indicator increases every month after the intervention, it means that the hospitals have an increasing demand for LCMs and pharmaceutical companies meet the demand as well. This indicator is from the demand side.

#### Monthly average delivery time

The monthly average arrival time of LCMs means the average time cost from hospitals sending the LCMs orders in the centralized bidding procurement and supply chain system (CBP system) to hospitals receiving the LCMs each month. A shorter time of delivery means LCMs could be transferred timely and would not likely contribute towards a shortage. This indicator is important to reflect the opportuneness of and the distribution capabilities of LCMs supply.

### Data source

The data were collected from the centralized bidding procurement and supply chain system (CBP system) [28]. The CBP system in Shandong was established in 2009 and it fully covered Shandong by the end of 2011 [29]. The system contains purchasing data of all medicines from the public hospitals in Shandong province [29]. Each piece of medicine procurement data contains detailed purchasing information of one medicine. The information includes the name of the region, the name of the buyer, procurement date, receiving date, medicine name, strength, dosage, package, the pharmaceutical company, request amount, and unit price [28].

We collected all LCM procurement data of secondary (*n* = 338) and tertiary public hospitals (*n* = 143) in Shandong province each month between April 2014 and March 2017. Our study finally consisted of 226,044 pieces of medicine procurement data including 1494 LCMs.

### Data analyses

#### Average Price quarterly and drug Price index analysis

The quarterly average prices are the average minimum unit prices of all LCM products purchased in one quarter. The minimum unit prices should be calculated according to medicines’ packages, for example, if the price of a bottle of medicine with 20 pills is 20 RMB, the minimum unit price of this medicine is 1 RMB. Although this indicator might not reflect the actual price level of LCMs, the trend of average prices could compare with the drug price index and give us the overall trend of price changes.

The types of drug price indexes are Laspeyres Price Index (I_L_), Passche Price Index (I_P_), Marshall-Edgeworth Price Index (I_M_) [30, 31]. The formulas are as follows:

$$ {I}_L=\frac{\Sigma {\mathrm{p}}_1{\mathrm{q}}_0}{\Sigma {\mathrm{p}}_1{\mathrm{q}}_0};\kern0.5em {I}_P=\frac{\Sigma {\mathrm{p}}_1{\mathrm{q}}_1}{\Sigma {\mathrm{p}}_1{\mathrm{q}}_0};\kern0.5em {I}_M=\frac{\Sigma {\mathrm{p}}_1\frac{{\mathrm{q}}_0+{\mathrm{q}}_1}{2}}{\Sigma {\mathrm{p}}_1\frac{{\mathrm{q}}_0+{\mathrm{q}}_1}{2}}; $$

The p means the average price of medicines during one period and q means the total quantity of medicine during one period. The p_1_ means the period we want to calculate the index and p_0_ means the period before p_1_ [32].

We calculated those three price indexes quarterly by set the first quarter (2nd quarter 2014) as a fixed q_0_ and p_0_ to compare the trend. If the trends of those three indexed are the same, we will use I_M_ and quarter-on-quarter I_M_ to reflect the price change. We did not consider Inflation index because there was no obvious inflation from 2014 to 2017 in China based on the data in world bank [33].

#### ITS analysis

A single ITS was conducted to evaluate the impact of LCM policy on medicine supply. The model is specified as followed:

$$ {\mathrm{Y}}_{\mathrm{t}}={\upbeta}_0+{\upbeta}_1\cdotp {\mathrm{T}}_{\mathrm{t}}+{\upbeta}_2\cdotp {\mathrm{X}}_{\mathrm{t}}+{\upbeta}_3\cdotp {\mathrm{X}}_{\mathrm{t}}{\mathrm{T}}_{\mathrm{t}}+{\upvarepsilon}_{\mathrm{t}} $$

Y_t_ is the aggregated outcome variable measured at each equally spaced time point t, in which is the monthly number of products, monthly procurement volumes, and monthly average delivery time of LCMs. T_t_ is the time since the start of the study, X_t_ is a dummy (indicator) variable representing the intervention (preintervention periods 0, otherwise 1), and X_t_T _t_ is an interaction term [34].

Using segmented linear regression, we could get the values of the pre-intervention slope, post-intervention slope, change in slope, and level change in the intervention. The pre-intervention slope is the increase/ decrease rate before the intervention, and the post-intervention slope is the increase/ decrease rate after the intervention. Therefore, the change in slope is the difference between the two. If the change in slope is positive, it means the indicator is increasing after the intervention and if negative, it shows it is decreasing. Level change in the intervention is the immediate change of indicator right after the intervention. The positive value means the indicator increased immediately once the intervention started. We used Stata/MP 14.0 for all analyses [34].

## Results

### The effects on prices

**Fig. 1:**
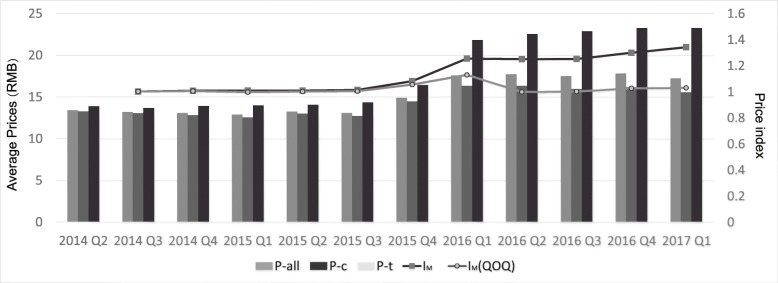
The trend of average prices and Drug Price Index of frequently-used LCMs by quarter, in Shandong Province, China Note: I_M_: Marshall-Edgeworth Price Index; I_M_ (QOQ): quarter-on-quarter Marshall-Edgeworth Price Index; P: average price of all LCMs; P_C_: average price of chemical LCMs; P_T_: average price of traditional Chinese LCMs

Before the LCM policy, prices of LCM products fluctuated with time. After the policy implementation in October 2015, the quarterly average price of LCM products showed a sudden increase, especially traditional Chinese medicines. After the two-quarter implementation of policy, the prices increase trend became smooth as before. Besides, overall, the average price of medicines is much lower than the average price of traditional Chinese medicines (Fig. 1).

Comparing average price of 2nd quarter 2014 with the 1st quarter 2017, there were 87.4% (*n* = 292) LCMs’ prices increase, 10.5% (*n* = 35) decrease and 2.1% (*n* = 7) stayed about the same. Here are some examples in Table 1 to show the significant price increase in the case of some LCM products.

**Table 1:** Examples of the LCM products with significant rising prices compared 1st quarter of 2017with 2nd quarter of 2014

Product names	Dosage per unit	Types^a^	Unit prices in 2014 Q2	Unit prices in 2017 Q1	Growth rate
Muxiang Shunqi pills	0.06 g	t	0.06	5.67	87.30
Baizi Yangxin Pills	0.1 g	t	0.09	2.47	25.17
Niuhuang Jiedu Tablets	none	t	0.03	0.58	20.90
Clotrimazole cream	10 g	c	0.50	18.00	35.00
Citrate spray tablets	25 mg	c	0.01	0.30	25.79
Esomezol tablets	1 mg	c	0.02	0.39	23.17

The price is in the form of RMB. ^a^The t in Types represents Traditional Chinese medicines, and c represents chemical medicines

The price index of those 334 LCM products shows that the results of Laspeyres Price Index (I_L_), Paasche Price Index (I_P_), and Marshall-Edgeworth Price Index (I_M_) have the same trend (Tab 2). Therefore, we use I_M_ to analyze the price change of LCMs. Figure 1 illustrates that the trend of LCMs’ price is continuously rising and has a rapid growth during the 3rd quarter, 2015 to 1st quarter, 2016. This is consistent with the average prices.

**Table 2:** The average prices and Drug Price Index of frequently-used LCMs by quarter, in Shandong Province, China

	2014 Q2	2014 Q3	2014 Q4	2015 Q1	2015 Q2	2015 Q3	2015 Q4	2016 Q1	2016 Q2	2016 Q3	2016 Q4	2017 Q1
I_L_		1.004	1.008	1.012	1.009	1.017	1.100	1.288	1.284	1.280	1.328	1.372
I_P_		1.001	1.007	1.006	1.007	1.011	1.067	1.229	1.224	1.231	1.283	1.314
I_M_		1.002	1.008	1.009	1.008	1.014	1.082	1.255	1.250	1.251	1.300	1.341
I_M_ (QOQ)		1.002	1.006	0.997	1.002	1.004	1.055	1.130	0.999	1.003	1.027	1.031
P-all	13.42	13.21	13.07	12.89	13.25	13.08	14.90	17.59	17.74	17.52	17.83	17.25
P-c	13.26	13.06	12.82	12.56	13.01	12.73	14.46	16.33	16.34	15.95	16.21	15.58
P-t	13.89	13.68	13.92	13.98	14.07	14.36	16.44	21.81	22.55	22.87	23.27	23.28

The price is in form of RMB

*I*_*L*_ Laspeyres Price Index; *I*_*P*_ Passche Price Index; *I*_*M*_ Marshall-Edgeworth Price Index; *I*_*M*_ (QOQ): quarter-on-quarter Marshall-Edgeworth Price Index; P-all: average price of all LCMs; P-_C_: average price of chemical LCMs; P-t: average price of traditional Chinese LCMs

### The effects on the supply

**Fig. 2:**
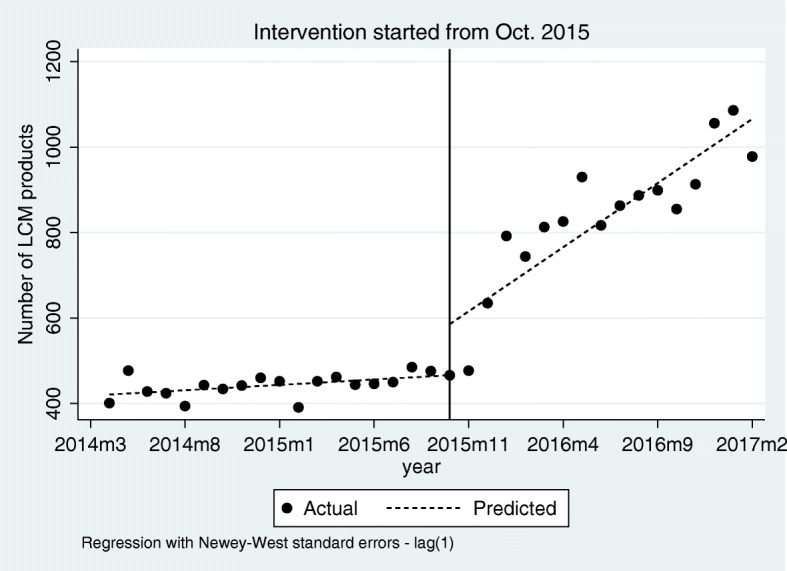
The effects of LCM policy on the number of LCM products before and after the policy intervention in October 2015, Shandong Province, China

There was a total of 1494 products of LCMs available in the Shandong provincial from April 2014 to February 2017. As for the monthly number of products supplied, there was a slight increase of 0.63% per month (Value = 2.51, *P* = 0.022) before the intervention. Only 31.19% (*n* = 466) of them were available in October 2015 when the LCM policy was initially implemented. After the 16-month implementation of LCM policy, in February 2017, the LCM products availability increased by 109.87% (*n* = 978, 65.46% of the total). The LCM policy made the monthly number of products increased significantly (value = 27.5, *P* < 0.001), and increased at a rate of 6.44% per month (Value = 30.02, *P* < 0.001) (Fig. 2, Table 3).

**Table 3:** Estimated coefficients of the segmented regression model for the monthly number of products, procurement volumes, and average delivery time of LCMs before and after the LCM policy intervention in October 2015, Shandong Province, China

Parameter	Value (SE)	t	P	[95% Conf. Interval]
**Number of products types**				
Intercept	420.92 (11.39)	36.99	< 0.001	[397.71, 444.13]
Pre-intervention slope ^a^	2.51 (1.04)	2.41	0.022	[0.38, 4.64]
Post-intervention slope ^c^	30.02 (5.38)	5.58	< 0.001	[19.04, 40.99]
Change in slope ^b^	27.50 (5.43)	5.07	< 0.001	[16.43, 38.57]
Level change in intervention	119.42 (60.21)	1.98	0.056	[−3.39, 242.23]
**Procurement volumes of all LCMs (packages)**				
Intercept	2,071,127.00 (117,796.50)	17.58	< 0.001	[1,830,880.00, 2,311,375.00]
Pre-intervention slope ^a^	19,771.37 (12,044.75)	1.64	0.111	[− 4794.06, 44,336.80]
Post-intervention slope ^c^	4.74e+ 05 (6.08e+ 04)	7.79	< 0.001	[350,000.00, 598,000.00]
Change in slope ^b^	454,101.60 (61,447.83)	7.39	< 0.001	[328,777.90, 579,425.30]
Level change in intervention	− 282,740.10 (382,843.10)	−0.74	0.466	[−10,635,540.00, 498,073.70]
**Procurement volumes of 154 LCMs (packages)**				
Intercept	1,905,371.00 (122,873.70)	15.51	< 0.001	[1,654,768.00, 2,155,973.00]
Pre-intervention slope ^a^	12,562.22 (11,348.67)	1.11	0.277	[−10,583.56, 35,707.99]
Post-intervention slope ^c^	2.42e+ 05 (3.70e+ 04)	6.52	< 0.001	[166,000.00, 317,000.00]
Change in slope ^b^	229,092.10 (38,127.01)	6.01	< 0.001	[151,331.50, 306,852.60]
Level change in intervention	−151,236.50 (292,263.30)	−0.52	0.609	[− 747,311.30, 444,838.30]
**Average delivery time of all LCMs (days)**				
Intercept	30.55 (0.91)	33.66	< 0.001	[28.70, 32.40]
Pre-intervention slope ^d^	−1.21 (0.07)	−16.66	< 0.001	[− 1.36, − 1.06]
Post-intervention slope ^e^	−0.08 (0.06)	−1.40	0.171	[−0.20, 0.04]
Change in slope ^b^	1.13 (0.09)	12.19	< 0.001	[0.94, 1.32]
Level change in intervention	1.08 (0.87)	1.24	0.223	[−0.69, 2.86]

SE, standard error

^a^ Indicates a non-significant rise in those indicators from month to month before the intervention

^b^ A significant change in the regression slope – indicating a significant increase in those indicators –was noted right after the intervention

^c^ indicates a significant rise was noted right after the intervention

^d^ indicates a significant decrease in average delivery time of LCMs from month to month before the intervention

^e^ indicates a non-significant rise or decrease in average delivery time of LCMs from month to month after the intervention

**Fig. 3:**
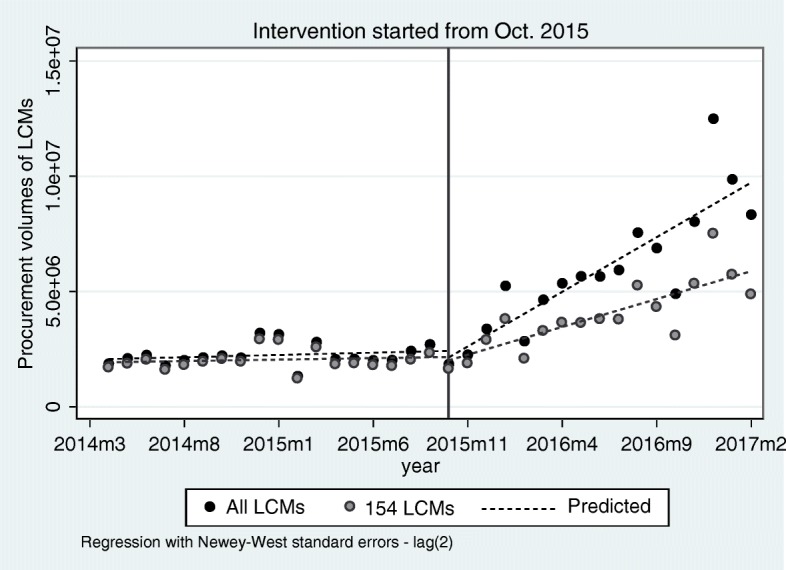
The effects of LCM policy on the monthly procurement volumes of LCMs (packages) before and after the policy intervention in October 2015, Shandong Province, China

As shown in Fig. 3 the trends of monthly procurement volumes of all LCMs and 154 LCMs are the same. Figure 3 shows that no significant increase in monthly procurement volumes of LCMs was noted before the intervention. Besides, the procurement volumes of 154 LCMs took up more than 90% of total procurement volumes of all LCMs before LCM policy. The pre-intervention slope remains stable. Furthermore, after the implementation of LCM policy in October 2015, the monthly procurement volumes of all LCMs increased rapidly, on average, at a rate of 28.93% per month for all LCMs (Value = 474,000, *P* < 0.001) and 13.06% per month (Value = 242,000, *P* < 0.001) for 154 LCMs. The intervention leads to a significant change in regression slope (*P* < 0.001) for monthly procurement volumes of all LCMs (Table 3).

**Fig. 4:**
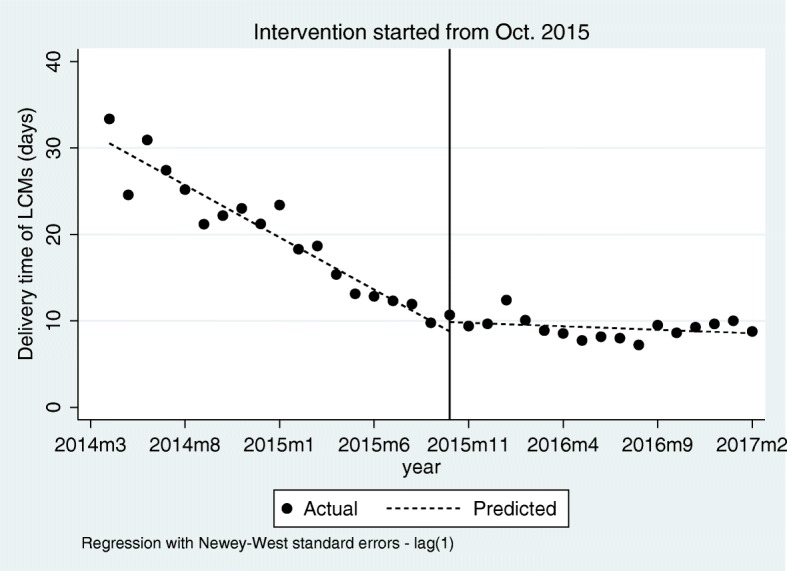
The effects of LCM policy on the average delivery time of LCMs (days) before and after the policy intervention in October 2015, Shandong Province, China

The average delivery time of LCMs kept on decreasing from 33.37 days to 10.69 days at a reduced rate of 3.63% (Value = − 1.21, *P* < 0.001) per month from April 2014 to October 2015 before the intervention. However, the LCM policy slowed down the reduction of delivery time and the change in the slope of delivery time increased by 1.13 (*P* < 0.001). No significant change in average delivery time was noted after the intervention and the delivery time of LCMs kept stable at 9 days on average (Fig. 4, Table 3).

## Discussion

The LCM policy had a positive impact on LCM supply and improved access to LCM for public hospitals. After the implementation of the LCM policy in October 2015, both the number of products and the volumes of LCMs increased significantly, which means the policy might promote the supply and the demand of LCMs. It also concludes that the LCM policy is beneficial to improve access to medicines. Besides the increasing trend is more obvious for traditional Chinese medicines and the reason might be that the traditional Chinese medicines have other incentives like policy support in industrial.

Nevertheless, the supply capacity of medicines needs to be strengthened [35] as the delivery time of LCM was 9 days, which was longer than 3 days – the time expected by policymakers of medicines procurement. Before the LCM policy, the government implemented policies like centralized procurement of medicines [36] to improve the distribution efficiency of medicines and the medicines delivery time was reduced. However, after the LCM policy, along with the increasing demands for LCMs, delivery time again increased. There are two possible explanations. One might be that the distribution capacity of pharmaceutical distribution firms has reached their limit and even though there is no LCM policy, the delivery time keeps sable at 9 days [37, 38]. Another explanation might be the LCM policy had a negative influence on distribution companies and the policy hindered the further decline of delivery time. However, this explanation needs further studies and evidence.

In our perception, LCMs are chiefly used in primary health institutes. However, it is worth noting that the LCM policy did not include primary health institutes at first stage in Shandong [18]. This is because China has special policies for primary healthcare institutions. In China, primary health institutions are required to mainly use essential medicines whose prices are set by provincial centralized bidding [36, 37], which means that the prices are fixed from a provincial level and are much cheaper. We think primary health institutes are not included because this policy somehow increases the price of LCMs and the government needs to maintain the lowest prices at the grassroots level. Even though there is medicine shortage problem caused by unreasonable prices in primary health institutes, government tend to use other measures like national essential medicine policy [39] instead of pricing policy to guarantee the supply and utilization [36].

We argue that an appropriate price increase is necessary as this could alleviate the shortage due to long-term unadjusted price ceiling. Our results show that the supply of LCMs had a rise along with the prices, which is in line with the studies done by Zhang (2016) [19], Song (2018) [20] and Guan (2018) [21]. Besides, they found that the increase in the prices of LCMs did not significantly increase the burden of health and medicines expenditures.

However, as the policy set the price cap of those LCMs by maximum daily cost, some LCMs with a small daily dose, such as cream, may have an unreasonable price increase and further increase can have an impact on patients’ healthcare expenditures. With the increasing supply of LCMs, the close monitoring of LCM prices should be implemented in the future.

Additionally, although the LCM policy had promoted the supply of some generic medicines, it also had problems. It was noteworthy that China’s National Development and Reform Commission, who is responsible for the price setting, selected the medicines [17, 18], while the selection criteria were not specific and clear, especially the standard of calculating daily costs of traditional Chinese medicines. Besides, a simple daily-cost price cap could not solve the shortage of problems of all LCMs since every medicine has a different daily dose. The rational of list selection should be improved.

The findings of this study may help policy-making of improving medicine access as medicine shortages are a common problem shared by healthcare institutions in most countries around the world [40]. If the price cap leads to medicines shortage, a reasonable pricing policy could solve the problem effectively. This kind of strategy could be used in low- and middle-income countries to meet the demands of generic medicines when pharmaceutical companies have no incentives for the production of lower-priced medicines.

Even so, using a price policy for medicines to improve the medicines supply should be cautious because when government influences medicine prices, the market will change and will contribute towards a long-run impact on supply and in turn, the price [21]. It is crucial to figure out what reasons contributed to the shortage of medicines before initiating those policies as interventions.

The reasons of medicine shortages are complex. The origin of a drug shortage problem can be located at the supply and demand side. At the supply side, manufacturing problems such as manufacturing difficulties, unavailability of raw materials, quality issues, non-compliance with applicable regulatory and natural disasters are reported [41–43]. Besides, distribution and supply problems are other influencing factors, such as just-in-time inventories and inappropriate levels of stock, parallel distribution [44], quotas, rationing, and transportation issues [40, 41]. The supply side can be influenced by policy measures such as allocation and quality requirements [45]. Furthermore, as we have shown in our study, pricing policies could also result in product discontinuation, especially concerning those long-standing and lower-priced medicines [41]. Studies showed that apart from price capping, internal or external reference pricing, and tendering may affect patients’ access to medicines [44, 46–48].

Our study is methodologically strong. We use the drug price index and Interrupted time series analysis to provide the evidence for policy evaluation. We also use several different indicators to illustrate this study. The study involved a relatively large amount of observations (35 months) from a reliable database.

### Limitations

The study also has limitations. It was conducted in only one province in China, and the findings may not be generalized in the other parts of China. On the other hand, by focusing on a single province, we were able to gather good data. Besides, we did not include primary healthcare institutes because, at the early stage of the implementation, primary healthcare institutes did not include into the CBP system for LCMs. Further analysis of LCMs in primary health institutes level will be essential and important.

Although using the interrupted time series analysis method could evaluate the effect of policy intervention by building counterfactual, a comparison group will make the study design a standard quasi-experimental design and avoid bias caused by other policies like zero-profit margin policy for medicines. Further studies with suitable comparison groups will provide a higher level of evidence.

## Conclusions

On the whole, LCM policy recovered the production of some LCMs and increased the supply of LCMs. Accessibility to some LCMs was improved by the policy, which is beneficial to UHC. However, further policies should focus on to further reduce prices and to reduce the delivery time to improve the access of LCMs.

## Data Availability

The original datasets analyzed during the current study are not publicly available due to our confidentiality agreement with the Shandong CBP system but are available from the corresponding author on reasonable request.

## Abbreviations

LCM: Low-cost medicine
UHC: Universal health coverage
NDRC: national development and reform commission
NHC: National health commission
RMB: Renminbi
ITS: Interrupted time series
CBP System: centralized bidding procurement and supply chain system
I_L_: Laspeyres price index
I_P_: Passche PRICE INDEX
I_M_: Marshall-edgeworth price index
